# A Comparative Evaluation of the Efficiencies of Different Rotary File Systems in Terms of Remaining Dentin Thickness Using Cone Beam Computed Tomography: An In Vitro Study

**DOI:** 10.7759/cureus.61566

**Published:** 2024-06-03

**Authors:** Vivek P Vadera, Sandhya K Punia, Saleem D Makandar, Rahul Bhargava, Pradeep Bapna

**Affiliations:** 1 Conservative Dentistry and Endodontics, Darshan Dental College and Hospital, Udaipur, IND; 2 Conservative Dentistry and Endodontics, School of Dental Sciences, Health Campus Universiti Sains Malaysia, Kubang Kerian, MYS; 3 Conservative Dentistry and Endodontics, Pacific Dental College and Hospital, Udaipur, IND

**Keywords:** 2shape file, remaining dentin thickness, cone beam computed tomography, one curve file, trunatomy file

## Abstract

Aim: The aim of this study was to evaluate and compare the dentin thickness of the mesio-buccal canal of the lower first molar after canal preparation with three different rotary file systems using cone beam computed tomography (CBCT).

Methodology: TruNatomy (Dentsply Sirona, USA), 2Shape (Micro-Mega, France), and One Curve (Micro-Mega, France) were the three different rotary files that were employed. A total of 45 excised human permanent first mandibular molars were divided into Groups A (TruNatomy), B (2Shape), and C (One Curve) at random. To measure the residual dentin thickness at 3 mm, 5 mm, and 7 mm from the radiographic apex, the mesial root of the tooth was removed from the tooth, and a mesio-buccal canal was taken. Samples were mounted in clear acrylic resin and were subjected to a pre-instrumentation CBCT scan. The mesio-buccal canal was cleaned and shaped while maintaining the final mesio-buccal canal preparation of Group A - 26/0.04, Group B - 25/0.04, and Group C - 25/0.04. The samples were extensively irrigated with 3% sodium hypochlorite and 17% EDTA, and a post-instrumentation scan was performed on them. Statistics were used to determine the values from CBCT scans that were recorded for pre- and post-instrumentations.

Results: The results showed that Group A had the greatest drop in dentin thickness, followed by Group B and Group C. The change in dentin thickness was greatest at 3 mm and 7 mm.

Conclusion: In contrast to TruNatomy and 2Shape rotary file systems, One Curve has the advantage of maintaining a tooth's thickness at 3 and 7 millimeters from the radiological apex. Since the TruNatomy file system removes more dentin than the other two combined, it should be used cautiously. Choosing the right instrument is crucial for cleaning and shaping during root canal preparation.

## Introduction

The removal of infected structures like infected tissue, microbes, smear layer, and necrotic and softened dentin from the root canal system is a crucial step in root canal treatment. This is done in order to obtain a consistent taper to make it easier to obliterate the prepared canal space [1]. However, excessive dentin removal during the canal preparation process could result in tooth issues such as strip perforation and vertical root fracture. The most important component in preventing root fractures, particularly vertical root fractures, is the amount of remaining dentin thickness(RDT) [2].

In order to remove dentin during shaping to predetermined limits that could not damage the root, mechanical instrumentation restrictions are dictated by residual dentin thickness [3]. The root's circumferential dentin wall thickness is significant because it has a direct bearing on the tooth's capacity to withstand lateral forces and avoid fracture [4]. After a root canal procedure, the amount of dentin that is still present is directly related to how hard the root is. A fracture-prone tooth is one with insufficient dentin [5]. To prevent root fracture following intra-radicular operations, all root aspects should have at least 1 mm of root dentin [6].

When using K-type files and reamers, conventional techniques included linear filling and rotational motions. When using traditional hand instrumentation, the root canal was first created from the apical constriction to the orifice after the working length had been measured radiographically. But because it is challenging to pre-bend stainless steel files with broad size to be used in curved canals, this leads to problems like straightening or deviation from the canal's original anatomy, which results in an excessive amount of dentin being removed from the wall, away from the center at apical region and from the wall close to the center of the tooth in the coronal region [7].

The inner walls of, the mesial roots of lower molars and buccal roots of maxillary molars are referred to as danger zones and are more prone regions for strip perforations. The lateral perforation is a result of extensive instrumentation within the weaker root wall. The "anti-curvature filing" technique was devised to prevent these accidents [8]. To facilitate access to the middle and apical regions, several authors advised flaring the coronal portion of the root canal [9]. Strip perforations and vertical root fractures are, however, caused by excessive flare, which lowers the root's resistance to fracture [10].

Traditional hand files were replaced by nickel-titanium (Ni-Ti) rotary devices because they need less chairside time for cleaning and shaping, improve the ability of the root canal to be shaped, and prevent clinical issues such as ledges, transportation, and perforation [11]. The single-file design uses just one Ni-Ti instrument, which lowers costs, shortens chair side time, and eases the operator's learning curve. An alloy that has been heat treated is more prone to plastic deformation, hampered cutting edges, and decreased cutting ability [12].

Dentsply Maillefer introduced the TruNatomy file system (Dentsply Sirona, USA) because of its greater capacity to centre canals and modify them based on canal anatomy. The files are composed of unique Ni-Ti-heated wire with an off-centered cross-section, which gives the file extra flexibility and enables pre-bending. However, TruNatomy is a multiple-file system that emphasizes the necessity for a system with fewer files because it necessitates more chair-side time [13].

Due to its increased flexibility, cyclic fatigue resistance, respect for the natural root canal architecture, and tripe helix cross-section, which strikes the perfect balance between cutting efficiency and debris removal, Micro Mega created the 2Shape file system (Micro-Mega, France) [14]. The Micro Mega "One Curve File System" (Micro-Mega, France) is a single file system that simplifies instrument management, prevents cross-contamination since it is designed for single use, and requires less chair side time for cleaning and shaping procedures. It is constructed of heat-treated Ni-Ti C-wire that has regulated memory, is pre-bendable, and preserves the curvature of the root canal. Excellent cutting efficiency and a centred trajectory are made possible by the changeable cross-section of files [15].

In order to assess the efficacy of tools and methods for root canal preparation, many techniques have been used to determine the form of canals before and after preparation. A variety of techniques have been utilized, including radiography, stereomicroscopy, computed tomography (CT) scans, cone beam computed tomography, and micro-computed tomography. CBCT imaging is a non-destructive technique among several mentioned in the literature for evaluating how well different tools and prepping methods can shape objects [16]. It regularly helps dental professionals by offering high-quality three-dimensional (3D) images of dental structures because of its exceptional spatial resolution. It can, therefore, be used to compare the remaining dentin thickness between instruments before and after utilizing various filing systems.

Therefore, the current study's goal was to evaluate, using CBCT, how well the TruNatomy, 2Shape, and One Curve file systems maintained the remaining dentine thickness. Research null hypotheses as there was no significant difference in the remaining dentin thickness when root canals were prepared with TruNatomy, 2Shape, and One Curve file systems.

## Materials and methods

For this study, 45 human permanent lower first molar sound teeth were taken from the Department of Oral and Maxillofacial Surgery, Darshan Dental College and Hospital, Udaipur, Rajasthan, India, which were extracted due to periodontal disease. the ethical approval was obtained from the Darshan Dental College and Hospital with approval no. DDCH/ADM2019/20/1301-Cons. The teeth included were sound teeth with visible and non-calcified canals. The study eliminated teeth with endodontic treatment, dental anomalies and resorption, calcified canals, and root curvature of more than 30°. The teeth chosen were cleaned and then preserved in ordinary saline until use. All of the teeth were decoronated at the cementoenamel junction (CEJ) level, separating the mesial roots from the distal roots using a diamond disc under water cooling. The mesio-buccal (MB) canal was chosen for the experiment. The air-rotor handpiece with Endo access and Endo Z bur from Dentsply Maillefer-USA was utilized to get access to the specimens' roots, which were calibrated to be 12 mm long. The canal's patency was assessed using a #10 stainless steel hand K-file (Dentsply Maillefer-USA) and the golden method; the file was inserted into the canal till the file was visible from the outer surface at the apex. The working length (WL) was determined just short of the apex at 0.5 mm. Canal curvature was assessed using the Schneider method, and roots with less than 30° of curvature were chosen. All selected specimens were embedded into a clear acrylic resin (DPI® RR) block (5.5×1.5×1.7 cm) with five roots in each model, and then 45 teeth roots were separated into three experimental groups, each with 15 roots: Group A - TruNatomy, Group B - 2Shape (T-wire shaping files), Group C - One Curve.

The following scanning settings were used for cone beam computed tomography (CBCT) scans for both pre-instrumentation before shaping with Ni-Ti files and post-instrumentation after shaping with Ni-Ti files as according to respective groups, scans: Fov-85, kVp-90, mA-4.0, and voxel-150×150×150 m. Exposure-time 15 s and CS3D Imaging software from Care Stream, USA, were utilized to calculate RDT.

With a #20 stainless steel hand K file, each root canal was widened and traversed. The root canal irrigation was followed by the use of 3% NaOCl and 17 % EDTA (smear layer removal and lubrication) during the cleaning and shaping of the canal. Complete chemo-mechanical preparation was carried out between the two irrigants using three distinct rotary file systems: Group A: TruNatomy file system- Canals were prepared till 26/0.04; Group B: 2Shape file system- Canals were prepared till 2Shape TS1, 25/0.04; Group C: One Curve file system- Canals were prepared until 25/0.04.

**Figure 1:**
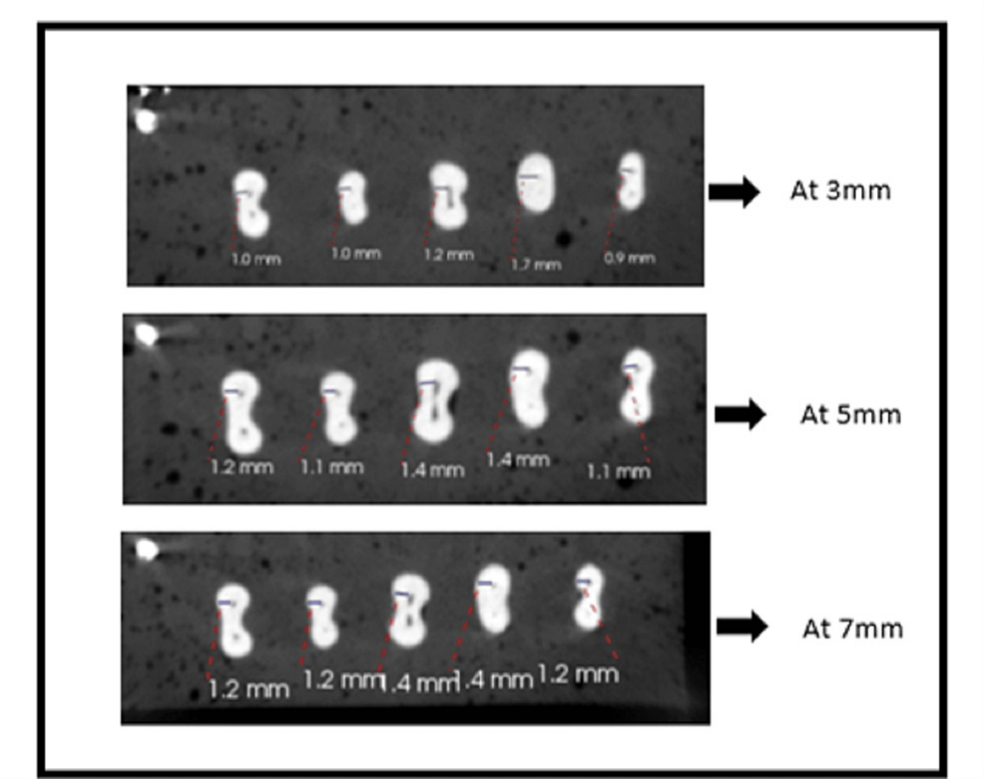
Pre-instrumentation CBCT image The cone beam computed tomography (CBCT) shows dentin thickness at 3 mm, 5 mm, and 7 mm pre-instrumentation.

**Figure 2:**
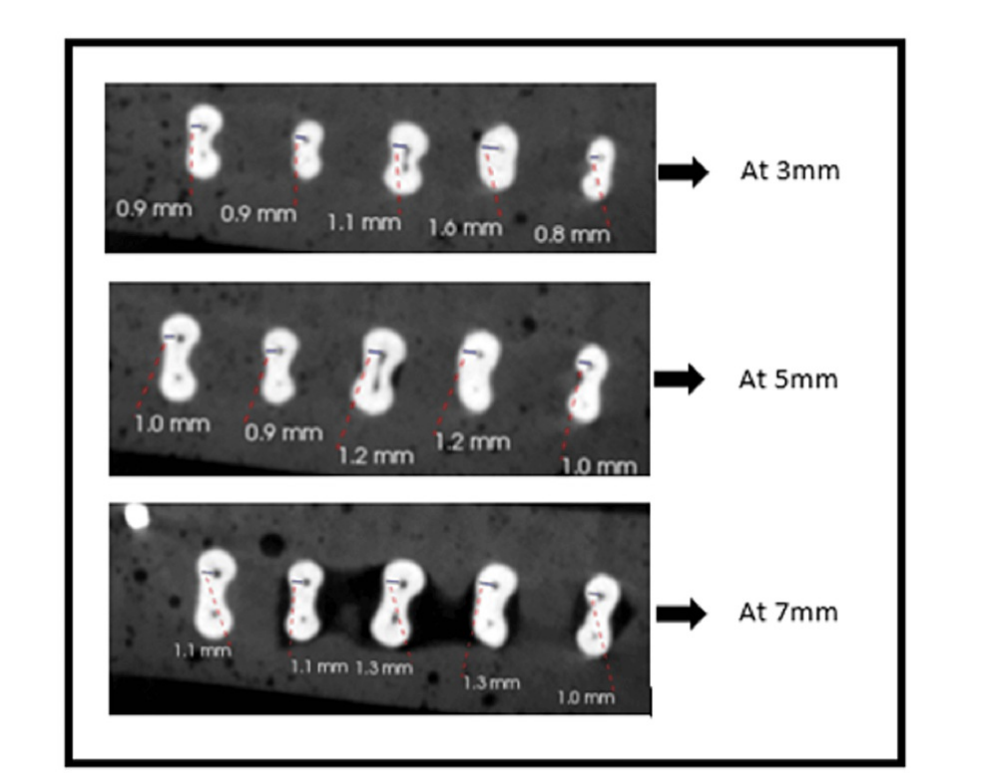
Post-instrumentation CBCT images The cone beam computed tomography (CBCT) images show dentin thickness at 3 mm, 5 mm, and 7 mm post-instrumentation.

Following the usage of the five MB models, a new file system was utilized for each group. The specimens were then exposed to CBCT (Carestream Kodak CS8100 3D) post-instrumentation scanning utilizing the same scanning parameters as those for pre-instrumentation scans after chemo-mechanical preparation with rotary files. For all specimens, pre- and post-instrumentation images were obtained in the axial section using the CS3D Imaging Software at distances of 3 mm, 5 mm, and 7 mm from the radiological apex (Figures 1, 2). The corresponding values for each group were entered into the computer. Pre-instrumentation dentin thickness and post-instrumentation dentin thickness were compared among the group to evaluate the file system that removes the least dentin from the MB canal of permanent mandibular first molar amongst the used rotary file systems.

The root length was marked using the software's available marking tool at 3 mm, 5 mm, and 7 mm per time, respectively, in the vertical and transverse tooth sections. Then, in the axial sections of the tooth, at the designated lengths, the marker was placed starting from the radiological apex. The dentin thickness was marked in the axial section starting from the center of the canal/outer lining of the canal until the dentin and cementum were differentiated. Pre- and post-instrumentation measurements for each tooth at 3 mm, 5 mm, and 7 mm were taken in the same way. The data was compiled and statistically analyzed using one-way ANOVA and repeated-measures ANOVA to determine the percent change in dentin thickness. Post hoc Tukey tests were used to assess the theme and differences in the tested groups.

## Results

**Figure 3:**
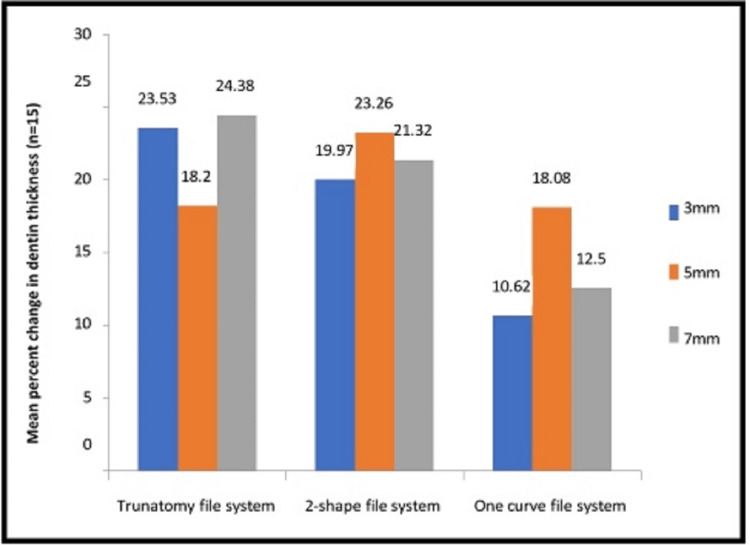
Comparative assessment of mean percent change in dentin thickness at 3 mm, 5 mm and 7 mm from apex among the study groups Data presented in mean percent change in dentin thickness at 3 mm, 5 mm, and 7 mm from apex (n=15).

Percentage change in the dentin thickness was observed maximum at 3 mm and 7 mm. Group A had thinner dentin than Group B or C (Table 1 and Figure 3). While Group B was discovered to be non-significant with a p-value of >0.253, Group A and Group C were determined to be statistically significant (Table 2).

**Table 1:** Comparative assessment of pre- and post-instrumentation dentin thickness at 3 mm, 5 mm and 7 mm from apex among the study groups The data has been represented as Mean±SD, p-value is considered significant at p<0.05.

Groups	Location from apex	Dentin thickness (Mean±SD)			
		Pre-instrumentation (n=15)	Post-instrumentation (n=15)	Change in thickness	Percent change in thickness (n=15)
A	3mm	1.04 ± 0.27	0.81 ± 0.27	0.23 ± 0.07	23.53 ± 8.72
	5mm	1.25 ± 0.31	1.04 ± 0.32	0.21 ± 0.05	18.20 ± 7.22
	7mm	1.44 ± 0.35	1.11 ± 0.34	0.33 ± 0.07	24.38 ± 8.14
B	3mm	1.04 ± 0.19	0.84 ± 0.21	0.20 ± 0.05	19.97 ± 6.62
	5mm	1.22 ± 0.15	0.94 ± 0.16	0.28+0.07	23.26 ± 7.09
	7mm	1.31 ± 0.18	1.04 ± 0.21	0.27 ± 0.07	21.32 ± 6.67
C	3mm	1.19 ± 0.24	1.06 ± 0.23	0.12 ± 0.05	10.62 ± 3.78
	5mm	1.34 ± 0.21	1.10 ±0.21	0.24 ± 0.08	18.08 ± 6.32
	7mm	1.45 ± 0.22	1.27 ± 0.22	0.18 ± 0.06	12.50 ± 4.99

**Table 2:** Comparative assessment of mean percent change in dentin thickness at 3 mm, 5 mm and 7 mm from apex among the study groups using one-way ANOVA The data has been represented as Mean±SD, p-value is considered significant at p<0.05.

Study groups	Percent change in dentin thickness (Mean±SD)			p-value (p<0.05)
	3mm ((n=15))	5mm ((n=15)	7 mm (n=15)	
GROUP A	23.53 ± 8.72	18.20 ± 7.22	24.38 ± 8.14	0.031*
GROUP B	19.97 ± 6.62	23.26 ± 7.09	21.32 ± 6.67	0.253
GROUP C	10.62 ± 3.78	18.08 ± 6.32	12.50 ± 4.99	0.001*
p-value (p<0.05)	0.000*	0.075	0.000*	

In Group A, the minimum dentin thickness was 5 mm (18.20±7.22), while in Group C was 3 mm (10.62±3.78). A post-hoc Tukey analysis revealed that Groups A, C, and B showed statistically significant differences in the change of the residual dentin thickness (Table 3).

**Table 3:** Inter-group comparison using Post hoc Tukey´s test

Groups	3 mm		5 mm		7 mm	
	Mean difference (n=15)	p-value (p<0.05)	Mean difference (n=15)	p-value (p<0.05)	Mean difference (n=15)	p-value (p<0.05)
GROUP A-GROUP B	3.55	0.322	-5.05	0.122	3.06	0.432
GROUP A-GROUP C	12.91	0.000*	0.12	0.572	11.88	0.000*
GROUP B-GROUP C	9.35	0.001*	5.18	0.111	8.81	0.002*

According to the statistical analysis, Group A (TruNatomy files) experienced the greatest reduction in dentin thickness and Group C (One Curve file) experienced the least reduction at 3 mm and 7 mm, respectively, whereas at 5 mm and between the tested groups, there was no statistically significant difference.

## Discussion

A number of features of the newly marketed endodontic instruments with different instrument designs need to be investigated and evaluated for efficiency in order to allow for efficient and secure clinical usage [17]. When used for cleaning and shaping, stainless steel file systems have the disadvantage of removing too much dentin, particularly in the apical region. In contrast to stainless steel instruments, Ni-Ti instruments' exceptional shape memory, and highly flexible alloy allowed for advancements in taper and flute design. Additionally, increased taper combined with Ni-Ti alloy improved predictability when using rotational methods to create a continuous canal form.

Compared to stainless steel files, nickel-titanium files mitigate this disadvantage by creating bigger dents. The Ni-Ti S files (files with S-shaped cross sections) offer a safe endodontic treatment of canals with more curvature as they reduce instrument failure [18]. There are many ways to compare the efficiency of various instruments for preparing root canals, including plastic blocks [19], radiographic techniques, histological sections, serial sectioning, scanning electron microscope, and silicone impressions of instrumented canals. The use of CBCT in research is one of the most recent developments in medicine; this scientific tool has the potential to boost endodontic research as well.

The MB canal for this investigation was located in the middle third of the mesial root of the mandibular first molar, which features a distal surface concavity and a root thickness of 0.19 to 0.7 mm. The mandibular molars are hence easily susceptible to perforations. Along with perforations, increased instrumentation in the apical third of mandibula teeth may result in lessening of the remaining dentin thickness, weakening the tooth structure [20]. The remaining root dentin thickness was measured using CBCT, which offers a feasible and non-destructive method for assessment before and after cleaning and shaping. Cross-sectional and three-dimensional (3D) pictures provided by CBCT are very accurate and measurable. Enough information is provided to compare the thickness of the brain before and after instrumentation [21,22]. When compared to other techniques for determining the thickness of a tooth, CBCT is more affordable and less invasive, which is one of its key advantages. Moreover, the radiation exposure in CBCT is lower than in Micro-CT [23].

To assess the effects of instrumentation along the canal's length, CBCT scans were carried out both before and after instrumentation. At three different levels, namely 3 mm, 5 mm, and 7 mm, the residual dentin thickness was measured from the radiographic apex of the root canal in an axial slice.

The remaining dentine thickness of the TruNatomy (Group-A), 2Shape (Group-B), and One Curve (Group-C) filesystems were compared in the current experiment. There were many studies available to evaluate the efficiency of various file systems in maintaining dentine thickness, with the exception of the study comparing them with various factors and metallurgy that was newly developed in the field of endodontics.

One Curve file system (Group-C) retained more dentin at 3 mm (10.62+3.78) and 7 mm (12.50+4.99) length from the radiological apex than the TruNatomy file system (Group-A), according to the results of this study's intergroup comparison of three file systems. The difference for all the tested groups was found to be statistically significant with p<0.05 except Group B.

Group C (One Curve file), the group that was used in this experiment, showed a positive reaction by maintaining more dentin thickness at 3 mm and 7 mm length from the radiography apex but significantly better dentin preservation at 7 mm. Dentin reduction was greatest in Group A (TruNatomy), followed by Group B (2 Shape), and Group C (One Curve). Despite the fact that the value at 5 mm for each group was deemed to be statistically insignificant because the p-value was more than 0.05, TruNatomy continued to have the highest dentin among the tested groups.

According to the literature, heat treatment of an alloy may make tools more prone to plastic deformation and disruption of cutting edges during use, reducing their capacity to cut. These instruments maintain a more central position in the canal because of the lower restoring force brought on by the proprietary heat treatment [12]. Less dentin may be extracted from the TruNatomy group at 5mm and One curve at 3 mm and 7 mm length from the radiographic apex of the root canal as a result. Additionally, due to the file system's orifice modifier having a 20/0.08 taper coronally, TruNatomy removed more dentin at a length of 7 mm. The better performance at 3 mm and 5 mm lengths could be attributed to the Onecurve file system, which is a single 25/0.04 file system.

In one study, there were no differences in apical transportation between TruNatomy (TN) and WaveOne Gold, Reciproc Blue, TRUShape , X P-endo Shaper, and iRace. Centering ability and canal transportation of One Curve (OC) files has limited research and documentation [24]. Razcha et al. compared the canal transportation and centering ability of OC, HyFlex EDM, HyFlex CM, and WaveOne Gold systems in moderately curved canals and they found no difference was noted between these systems in terms of centring ability. In parallel with the mentioned study, we found that the centering ability of One Curve (OC), TruNatomy (TN), and Protaper Next (PTN) systems were also similar. Razcha et al. also reported, at apical 3 mm and 5 mm sections there was no difference in canal transportation between OC and other systems, but transportation of OC in the lingual side was greater than HyFlex CM at 7 mm [25].

Tufenkçi et al. The shaping ability has been evaluated in resin blocks at angle of 45°, the apical transportation of canal at apical third of OC was less compared to PTN. At 5 mm and 8 mm, there was no difference between the files. Similarly, the OC file caused significantly less apical transportation than PTN, with no statistically significant difference between the files in the straight section of the ‘S’ shaped canals in resin blocks [26]. In this study, the ProTaper Next, One Curve, and TruNatomy instruments in curved root canals were used and centering ability and canal transportation were evaluated. No significant differences were observed between the groups or root canal levels in canal transportation and centering ability. The TruNatomy system demonstrated comparable results with both predecessors ProTaper Next and One Curve single-file systems [27].

According to the current study, even when all of the examined groups used files with the same tip and taper sizes, dentin removal varied between the groups even when the files were the same length. Analysis revealed that Groups A, C, and B showed statistically significant differences in the change of the residual dentin thickness. In contrast to Trunatomy and 2Shape rotary file systems, One Curve has the advantage of maintaining a tooth's thickness at 3 and 7 millimeters from the radiological apex. The order of the file system, cross-section, and metalworking all play a significant part in removing the dentin [28]. Along with the root canal curvature, endodontic mistakes are prevented in large part by curvature, which must be carefully assessed because, on radiographs, the curvature angle is never as great as the actual curvature angle of the root [29]. The following scanning settings were used for cone beam computed tomography (CBCT) scans for both pre-instrumentation before shaping with Ni-Ti files and post-instrumentation after shaping with Ni-Ti files as according to respective groups, scans: Fov-85, kVp-90, mA-4.0, and voxel: 150 x 150 x 150 m. Exposure time 15 s and CS3D Imaging software from Care stream-USA was utilized to calculate RDT and it followed the previous research [30,31]. Within the limitations of the present study, instrument selection plays an important role in minimizing unnecessary weakening of tooth structure, retaining the original shape of the canal, to maximize the cleaning effectiveness and to achieve the optimal results.

## Conclusions

In contrast to TruNatomy and 2Shape rotary file systems, One Curve has the advantage of maintaining a tooth's thickness at 3 mm and 7 mm from the radiological apex. Since the TruNatomy file system removes more dentin than the other two combined, it should be used cautiously. The dentin thickness plays a pivotal role in maintaining the strength of the tooth. Minimal preparation of the canal and achieving complete disinfection is the key to a successful treatment outcome. Hence, the remaining dentin thickness should be the most important factor when selecting instruments for cleaning and shaping of root canals to avoid root fractures and increase the treatment outcome. In our study, One Curve shows minimal dentin removal.

## References

[1] Rao MS, Shameem A, Nair R, Ghanta S, Thankachan RP, Issac JK (2013). Comparison of the remaining dentin thickness in the root after hand and four rotary instrumentation techniques: an in vitro study. J Contemp Dent Pract.

[2] Tabrizizadeh M, Reuben J, Khalesi M, Mousavinasab M, Ezabadi MG (2010). Evaluation of radicular dentin thickness of danger zone in mandibular first molars. J Dent (Tehran).

[3] Zuckerman O, Katz A, Pilo R, Tamse A, Fuss Z (2003). Residual dentin thickness in mesial roots of mandibular molars prepared with Lightspeed rotary instruments and Gates-Glidden reamers. Oral Surg Oral Med Oral Pathol Oral Radiol.

[4] Plotino G, Grande NM, Falanga A, Di Giuseppe IL, Lamorgese V, Somma F (2007). Dentine removal in the coronal portion of root canals following two preparation techniques. Int Endod J.

[5] Sathorn C, Palamara JE, Palamara D, Messer HH (2005). Effect of root canal size and external root surface morphology on fracture susceptibility and pattern: a finite element analysis. J Endod.

[6] Tomer AK, Miglani A, Chauhan P, Malik N, Gupta A (2016). Residual dentine thickness. Int J Appl Dent Sci.

[7] Elizabeth MS (2005). Hand instrumentation in root canal preparation. Endod Topics.

[8] Abou-Rass M, Jann JM, Jobe D, Tsutsui F (1982). Preparation of space for posting: effect on thickness of canal walls and incidence of perforation in molars. J Am Dent Assoc.

[9] Berutti E, Fedon G (1992). Thickness of cementum/dentin in mesial roots of mandibular first molars. J Endod.

[10] Schilder H (1974). Cleaning and shaping the root canal. Dent Clin North Am.

[11] Peters OA, Paque F (2010). Current developments in rotary root canal instrument technology and clinical use: a review. Quintessence Int.

[12] Yuan G, Yang G (2018). Comparative evaluation of the shaping ability of single-file system versus multi-file system in severely curved root canals. J Dent Sci.

[13] Van der Vyver PJ, Vorster M, Peters OA (2019). Minimally invasive endodontics using a new single-file rotary system. Int Dent Afr Ed.

[14] Faisal I, Saif R, Alsulaiman M, Natto ZS (2021). Shaping ability of 2Shape and NeoNiTi rotary instruments in preparation of curved canals using micro-computed tomography. BMC Oral Health.

[15] Adıgüzel M, Öztekin F (2020). Comparison of the resistance to cyclic fatigue of One Curve, One Shape, 2Shape and EdgeFile X3 files in simulated single and S-shaped (double) curvatures. Int Dent Res.

[16] Dhingra A, Ruhal N, Miglani A (2015). Evaluation of single file systems Reciproc, Oneshape, and WaveOne using cone beam computed tomography-an in vitro study. J Clin Diagn Res.

[17] Akhlaghi NM, Bajgiran LM, Naghdi A, Behrooz E, Khalilak Z (2015). The minimum residual root thickness after using ProTaper, RaCe and Gates-Glidden drills: a cone beam computerized tomography study. Eur J Dent.

[18] Weine FS, Kelly RF, Lio PJ (1975). The effect of preparation procedures on original canal shape and on apical foramen shape. J Endod.

[19] Weine FS, Kelly RF, Bray KE (1976). Effect of preparation with endodontic handpieces on original canal shape. J Endod.

[20] Iqbal MK, Banfield B, Lavorini A, Bachstein B (2007). A comparison of LightSpeed LS1 and LightSpeed LSX NiTi rotary instruments in apical transportation and length control in simulated root canals. J Endod.

[21] Inan U, Gonulol N (2009). Deformation and fracture of Mtwo rotary nickel-titanium instruments after clinical use. J Endod.

[22] Ghoddusi J, Bagherpour A, Mahmudabadi F, Forghani M, Sarmad M (2013). Residual dentin thickness of bifurcated maxillary premolars following two post space preparation methods. Iran Endod J.

[23] Michetti J, Maret D, Mallet JP, Diemer F (2010). Validation of cone beam computed tomography as a tool to explore root canal anatomy. J Endod.

[24] Pérez Morales ML, González Sánchez JA, Olivieri JG, Elmsmari F, Salmon P, Jaramillo DE, Terol FD (2021). Micro-computed tomographic assessment and comparative study of the shaping ability of 6 nickel-titanium files: an in vitro study. J Endod.

[25] Razcha C, Zacharopoulos A, Anestis D, Mikrogeorgis G, Zacharakis G, Lyroudia K (2020). Micro-computed tomographic evaluation of canal transportation and centering ability of 4 heat-treated nickel-titanium systems. J Endod.

[26] Tufenkci P, Orhan K, Celikten B, Bilecenoglu B, Gur G, Sevimay S (2020). Micro-computed tomographic assessment of the shaping ability of the One Curve, One Shape, and ProTaper Next nickel-titanium rotary systems. Restor Dent Endod.

[27] Hazar E, Geduk G, Coşkun E, Koçak S, Sağlam BC, Koçak MM (2023). Comparison of centering ability and canal transportation of TruNatomy files with different file systems. J Dent Indones.

[28] Khandagale PD, Shetty PP, Makandar SD (2021). Evaluation of cyclic fatigue of Hyflex EDM, Twisted Files, and ProTaper Gold manufactured with different processes: an in vitro study. Int J Dent.

[29] Makandar SD, Khaiser MI, Mali SR, Karobari MI, Marya A, Messina P, Scardina GA (2021). Plywood jig-a new technique for root canal curvature measurement. Appl Sci.

[30] Kamble AP, Pawar RR, Mattigatti S, Mangala TM, Makandar S (2017). Cone-beam computed tomography as advanced diagnostic aid in endodontic treatment of molars with multiple canals: two case reports. J Conserv Dent.

[31] Razumova S, Brago A, Barakat H, Serebrov D, Guryeva Z, Parshin GS, Troitskiy VI (2023). Evaluation of dentinal thickness and remaining dentine volume around root canals using cone-beam computed tomography scanning. Dent J (Basel).

